# A preliminary, observational study using whole-blood RNA sequencing reveals differential expression of inflammatory and bone markers post-implantation of percutaneous osseointegrated prostheses

**DOI:** 10.1371/journal.pone.0268977

**Published:** 2022-05-26

**Authors:** Andrew Miller, Sujee Jeyapalina, Jay Agarwal, Mitchell Mansel, James Peter Beck

**Affiliations:** 1 Research, George E. Wahlen Department of Veterans Affairs Medical Center, Salt Lake City, Utah, United States of America; 2 Division of Plastic and Reconstructive Surgery, Department of Surgery, School of Medicine, University of Utah, Salt Lake City, Utah, United States of America; 3 Department of Biomedical Engineering, University of Utah School of Engineering, Salt Lake City, Utah, United States of America; 4 Undergraduate Research Opportunities Program, University of Utah, Salt Lake City, Utah, United States of America; 5 Department of Orthopaedics, University of Utah School of Medicine, Salt Lake City, Utah, United States of America; Indiana University Purdue University at Indianapolis, UNITED STATES

## Abstract

**Aims:**

While the benefits of direct skeletal attachment of artificial limbs are well recognized, device failure due to infection and insufficient osseointegration remain obstacles to obtaining consistently successful outcomes. Currently, the potential for device failure is assessed by subjective pain, clinical function scores, radiographic evidence of bone atrophy, and the presence of radiolucent lines at the bone-implant interface, and subjective pain and function scores. Our hypothesis is that measurable biological indices might add another objective means to assess trends toward bone and stomal healing. This longitudinal cohort study was undertaken to identify potential serological biomarkers suggestive of bone remodeling and the presence of stomal tissue inflammation.

**Methods:**

Ten unilateral transfemoral amputee veterans, who were implanted with a percutaneous osseointegrated (OI) skeletal limb docking system, were recruited to participate in this IRB-approved study. Venous blood samples were obtained from before the Stage 1 Surgery up to 1 year following the Stage 2 Surgery. Whole-blood RNA was extracted, sequenced, mapped, and analyzed. Of the significant differentially expressed (DEGs) genes (p<0.05) identified, four genes of interest (IL12B, IL33, COL2A1, and SOST) were validated using qPCR. Enrichment analysis was performed to identify significant (p<0.01) Gene Ontology (GO) terms.

**Results:**

Most differentially expressed genes were only detected at PoS1 immediately after the first surgery. Of the significant genes identified, IL12B and IL33 were related to inflammation, and COL2A1 and SOST were associated with bone remodeling. These four genes were identified with greater than 20 log fold-change.

**Conclusion:**

Whole-blood RNA-seq data from 10 patients who previously underwent percutaneous osseointegrated lower limb implantation revealed four genes of interest that are known to be involved in inflammation or bone remodeling. If verified in future studies, these genes may serve as markers for predicting optimal bone remodeling and stomal tissue healing following OI device implantation.

## Introduction

**Fig 1:**
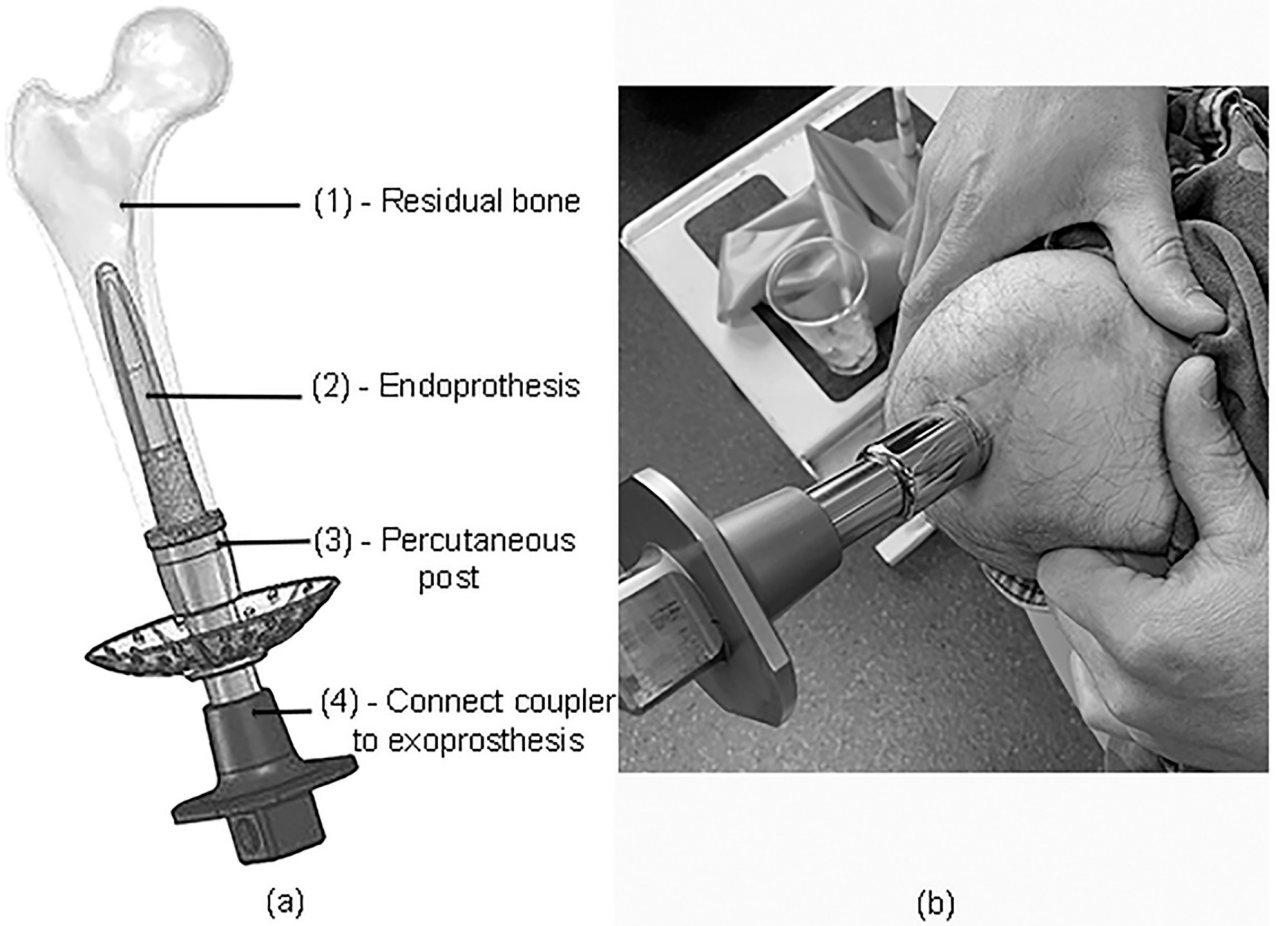
**(a)**—Diagrammatical representation of the Utah percutaneous osseointegrated device. An endoprosthesis (2) is inserted into the surgically prepared medullary canal of the residual femur (1), and a percutaneous post (3) is attached to the exoprosthesis using a Ferrier Coupler (4). (b)—A representative photograph shows the skin-implant interface of one study subject.

The direct attachment of an artificial limb to the residual stump bone requires that an implant traverse the overlying skin and become firmly osseointegrated (OI) within the medullary canal. This construct provides amputees with an optimum and robust exoprosthetic docking system (Fig 1). The benefits of this technology have been well demonstrated in multiple cohort studies [1–3]. Patients with limb loss, who have received percutaneous OI prostheses, have reported vastly improved upper and lower extremity prosthetic function compared to their prior use of conventional socket suspension systems [3–5].

Functional ambulation using skeletal docking devices requires that the adjacent endosteal bone in the medullary canal to become strongly osseointegrated within the structure of the implant surface. Over time, this integration depends upon the metabolic process of bone deposition, resorption, and remodeling. These processes occur continually in response to the loading and unloading forces applied to the skeletal bone and are described by “Wolff’s Law of bone adaption” [6]. In theory, if OI device design configurations support appropriate loading of the implanted bone, inducing robust bone formation around the implant and avoiding bone loss from “stress-shielding,” these percutaneous devices could last the lifetime of the recipient [7, 8]. However, some currently used implant systems fail to maintain permanent osseointegration because the design configuration intrinsic to the implant does not support bone deposition and over time encourages stresh shielded bone loss. Such resorption can also result in stomal collapse if the skin around the percutaneous post is attached to this underlying resorbing [9–12]. To date, there are no techniques that are capable of predicting early implant demise secondary to the failure of the process of osseointegration.

In addition to optimal osseointegration without distal resorption, there are legitimate concerns about the vulnerability of the stoma to superficial and potentially deep infection [2, 13]. It has been clinically established that a stable and mature stoma is vital to the longevity of these percutaneous OI devices. Although wound healing processes are known to be impaired around such implants [14], the material surface of the implant in contact with the tissue lining the stoma, must encourage completion of wound healing and avoid a continuous state of chronic inflammation at this interface. Currently, clinical observation and patient-reported outcomes are used to assess the completion of wound healing. As intimated above, objective quantitative techniques that non-disruptively detect the end of bone and stomal wound healing have not been developed.

Various invasive techniques have been used in animal models to study the local biological processes involved in these healing tissues [15–17]. However, in the clinical setting, due to both ethical and patient safety considerations, serial acquisition of local tissue is difficult, if not, impossible. In contrast, the analysis of whole-blood samples offers a non-invasive alternative that may be used to assess systemic biological processes of interest. Although multiple protein biomarkers have been correlated with bone-related diseases [18], such analyses have not been used as a predictive means for determining early indications of device success, impending failure, or the achievement of the final steady-state of complete wound healing. In contrast to typical assays used for protein detection in tissue samples, RNA sequencing (RNA seq) enables large-scale assessment of tissue activity via measurement of the transcriptome. Thus, RNA-seq was chosen as the preferred method for this investigation. The aim of this study was to use whole-blood RNA-sequencing to identify potential biomarkers involved in inflammation and bone metabolism, which, over time, might indicate the resolution of inflammatory wound healing processes, as well as the progression or completion of osseointegration. Once determined, these mRNA markers may be used to monitor the clinical progression of OI healing using relatively inexpensive PCR techniques.

## Methods

### Study design

Ten veteran unilateral transfemoral amputees, who were recruited to participate in an FDA-approved Early Feasibility Study of a POP device (NCT02720159), were also included in this current study (NCT02564432) with IRB approval (#00073178). The study design, described in detail previously [19], is briefly outlined below. A (Level II) prospective, longitudinal, observational study was conducted where participating patients underwent two separate surgical procedures to fit a transfemoral percutaneous OI implant [DJO global, Austin, TX] with a wait time of 6–8 weeks between procedures. The first procedure involved soft tissue revision, preparation of the residual bone to accept an appropriately sized endo-prosthesis, and device implantation. The second procedure involved coring the skin and muscle covering the implanted device and connecting an exo-prosthesis through the skin opening (i.e., stoma).

### Inclusion/Exclusion criteria

Patients were required to be within the US Veteran Health Care System with a unilateral, trauma-related, transfemoral amputation that was not a result of dysvascular disease. Patients were required to have used or be currently using a socket suspension prosthesis and the use of a non-propulsive, passive microprocessor-regulated lower limb prosthesis. Patients had to agree not to participate in high levels of physical activity while in the study.

Exclusion criteria included currently being on active military duty, the loss of more than one, the diagnosis of insulin-dependent or adult-onset diabetes, or a recent tobacco use history.

### Sample collection and processing

**Fig 2:**
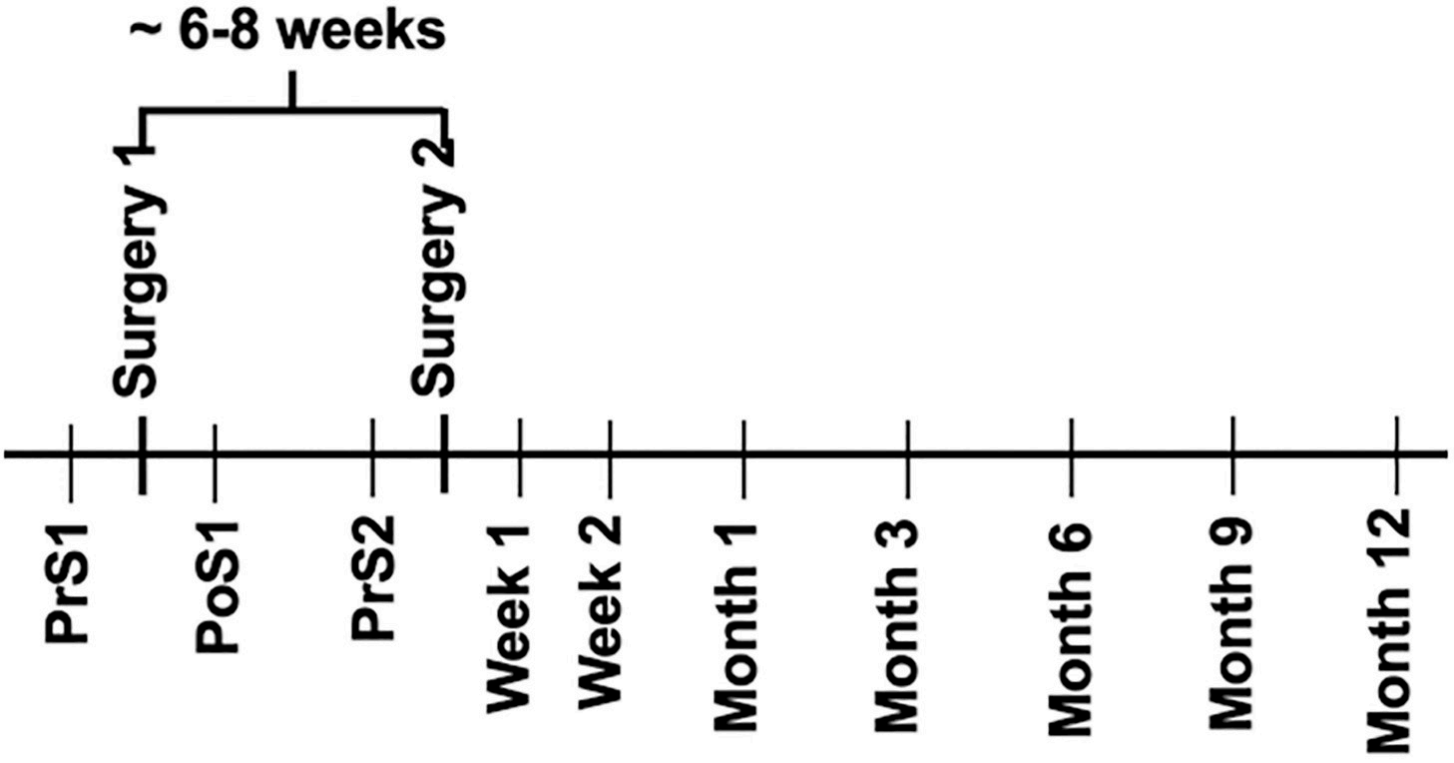
Two surgical procedures were performed 6–8 weeks apart. PrS1–1 week before Stage 1 Surgery; PoS1- one-week post Stage 1 Surgery; PrS2—one week prior to Stage 2 Surgery; PoS2-W1 -1-week post-Stage 2 Surgery; PoS2-W2–2 weeks post Stage 2 Surgery; PoS2-M1–1-month post Stage 2 Surgery; 3, 6, 9, and 12 months post Stage 2 Surgery are denoted as PoS2-M3, PoS2-M6, PoS2-M9, PoS2-M12, respectively.

Fifteen mL venous whole-blood samples were collected from patients at the following time points during the study (Fig 2): one week before Stage 1 Surgery (PrS1), one week post Stage 1 Surgery (PoS1), one week before Stage 2 Surgery (PrS2), 1-week post-Stage 2 Surgery (PoS2-W1), 2 weeks post Stage 2 Surgery (PoS2-W2), 1-month post Stage 2 surgery (PoS2-M1), and 3, (PoS2-M3), 6 (PoS2-M6), 9 (PoS2-M9), and 12 (PoS2-M12) months post Stage 2 Surgery, respectively. Total RNA was isolated from whole-blood samples using blood RNA stabilization tubes [PAXgene Blood RNA Kit, Qiagen, Valencia, CA]. Sample RNA quality was assessed using electrophoresis [Agilent RNA ScreenTape Assay, Agilent, Santa Clara, California]. Total RNA isolated from the venous blood samples underwent ribosomal depletion and library prep for sequencing [Illumina TruSeq Stranded RNA Kit with Ribo-Zero Gold, Illumina, San Diego, California]. Single-end 50bp sequencing was performed by the High-Throughput Genomics and Bioinformatics Analysis Shared Resource at the Huntsman Cancer Institute at the University of Utah, which was supported by the National Cancer Institute of the National Institutes of Health (Award Number P30CA042014). Sequence quality was assessed with MULTIQC v1.11 [20] incorporating RNA-seq metrics from FASTQC v0.11.9 [21].

### Sequence alignment and feature quantification

Samples were required to have an RNA Integrity Number (RIN) > 6 for inclusion. The resulting sequences were aligned to reference genome GRCh38.94 (STAR [22] via twopassMode). Feature quantification was performed using featureCounts v1.6.3 [23] with Ensembl annotations v94 to produce the feature count matrix.

### Count matrix pre-filtering

Because hemoglobin (HGB) sequences can make up a large portion of the RNA contained in whole-blood samples and has previously been shown to mask signals from lower expressed genes [24], HGB counts were identified bioinformatically using Ensembl annotations. Counts for the following HGB genes were removed: HBA1, HBA2, HBB, HBD, HBG1, HBG2, HBE1, HBM, HBQ1, HBZ. In order to be included for further analysis, count filtering was applied, which required at least 2 reads to be assigned to a gene in at least 5 samples.

### Differential gene expression analysis

The filtered count matrix was normalized using the default normalization function in DESeq2 v1.32.0 [25] before applying surrogate variable analysis (sva) [26]. The number of surrogate variables generated was five based on the “be” method from the sva package v3.40.0. Pair-wise comparisons were made between PrS1, which served as each patient’s baseline, and all subsequent time points using DESeq2 to test for differential expression. For each comparison, surrogate variables, patient, and time points were included in the DESeq2 statistical model. Multiple testing correction was performed using the Benjamini-Hochberg method with a false discovery rate (FDR) of 0.05.

### qPCR validation

Total RNA was extracted from whole blood samples [PAXGene RNA Extraction Kit, Valencia, CA] from three patients at time points PrS1 and PoS2-M3. Extracted RNA was reverse transcribed to cDNA [High-Capacity cDNA Reverse Transcription Kit, Thermo Fisher Scientific, Carlsbad, CA] combined with Power SYBR Green PCR Master Mix [Thermo Fisher Scientific, Carlsbad, CA] and forward and reverse primers for GAPDH, IL33, IL1RL2, COL2A1, and SOST (Table 1). The Ct values for all samples were normalized to GAPDH expression, and all plates were normalized using a common control. Fold-change was calculated using 2^−ΔΔCt^ values.

**Table 1:** Genes validated using qPCR with their respective primers.

Gene	Primer
IL-33	forward—GTGACGGTGTTGATGGTAAGAT
	reverse—AGCTCCACAGAGTGTTCCTTG
IL1RL2	forward—TACCACTCTGATTGTGGACTGC
	reverse—CCCCTTCTCTGATTCGTTTGGAT
COL2A1	forward—TGGACGCCATGAAGGTTTTCT
	reverse—TGGGAGCCAGATTGTCATCTC
SOST	forward—ACCACCATGGAGAAGGC
	reverse—GGCATGGACTGTGGTCATGA

### Enrichment analysis

The Enrichment analysis and associated visualizations were produced using the ViSEAGO v1.6.0 [27] R package. First, genes were annotated using the gene ontology (GO) [28] biological process annotations. The node size was set to 5, requiring at least 5 genes to be annotated to a GO term, and a Fisher’s Exact Test was then applied. Due to the overlap between genes assigned to separate GO terms and that multiple testing methods assume independence of tests, multiple testing correction was not applied in the enrichment analysis. Instead, topGO’s elim algorithm v2.44.0 [29] was used. When a node was found to be significant using the elim algorithm, the corresponding genes were marked and removed from all parent nodes prior to applying the Fisher’s Exact Test on parent nodes. A p-value below 0.01 was considered significant. To aid in the visualization and identification of relevant GO terms, the semantic similarity between significantly enriched terms was calculated using the method described by Wang et al. [30] Hierarchical clustering was performed and visualized using these similarity measures and the dynamicTreeCut 1.63.0 [31] package. The sequence data has been submitted to the Gene Expression Omnibus (GEO) under accession number GSE194108.

## Methods

### Study design

Ten male adult patients with unilateral trans-femoral amputations were recruited to participate in this IRB-approved study. Two patients experienced device failures. One patient sustained an accidental high-energy injury with a resulting periprosthetic femoral fracture eight months after the Stage 2 surgery. Prior to this unfortunate event, this patient achieved clinically robust osseointegration of the implant and a stable stoma. The second patient was removed from the study early on because of the failure of osseointegration and device loosening. Blood samples were not collected from these patients after the device failure.

### Sequence alignment and quantification

One patient was not available for follow-up at PoS2-M9 and after removing samples of low quality, 92 samples were included for downstream analysis (Table 2). On average, the selected samples had a RIN of 7.83. The mean base-pair quality scores were > 32, with an average GC content of 49%. Samples had, on average, 30.7 million reads aligned with 98.7% reads mapped (77.7% uniquely mapped reads, 21% multi-mapped reads). Ribosomal mapped reads were effectively removed during sample processing (0.0058% reads mapped to ribosomal locations).

**Table 2:** Total number of patient samples collected at the pre-selected time points.

Time Point	Number of Samples
PrS1	10
PoS1	10
PrS2	10
PoS2-W1	10
PoS2-W2	10
PoS2-M1	10
PoS2-M3	9
PoS2-M6	9
PoS2-M9	6
PoS2-M12	8

### Gene expression and enrichment analyses

**Fig 3:**
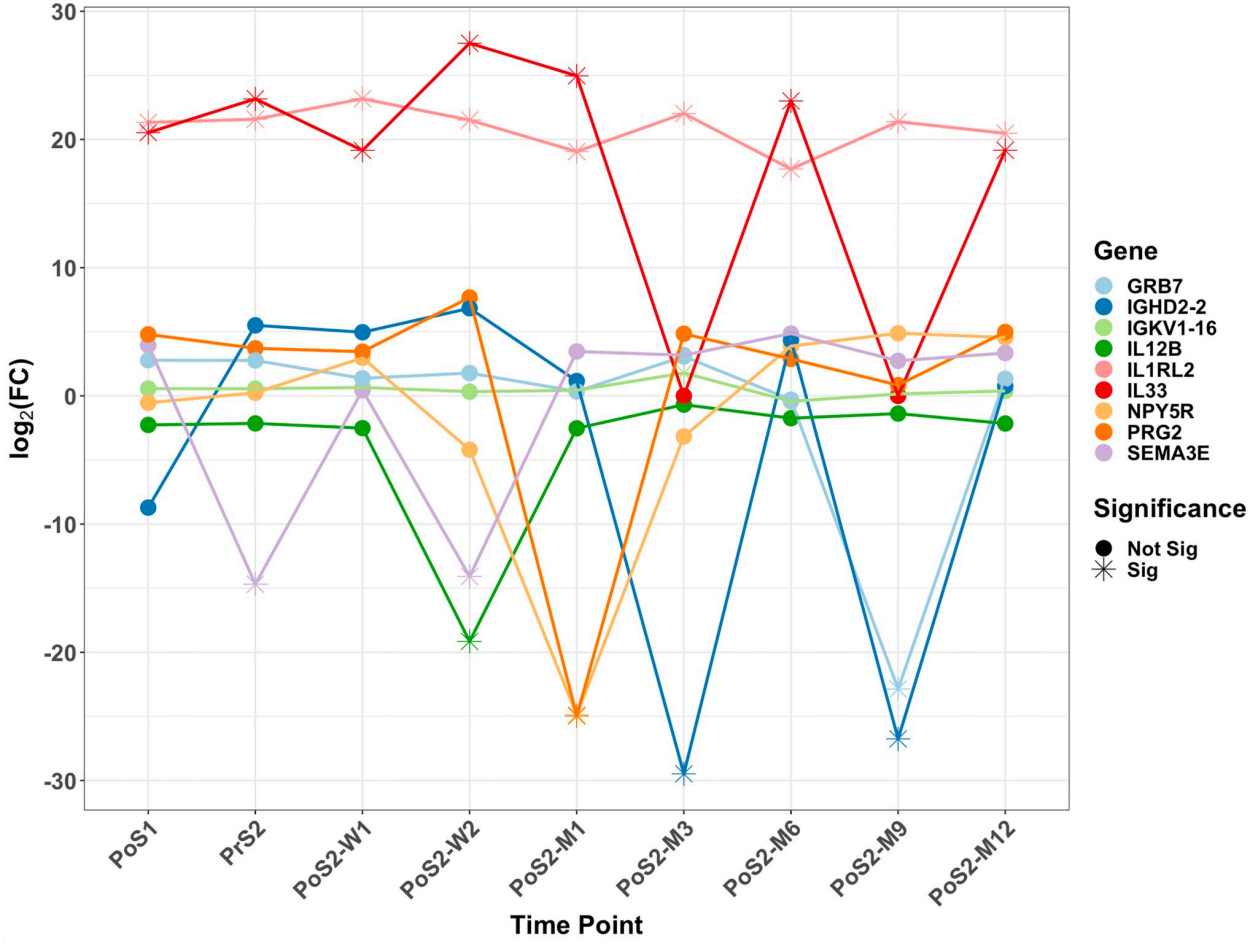
A scattergram showing the significantly differentially expressed genes annotated to inflammatory-related GO processes. Each time point was compared to the PrS1—which is considered as the baseline—read counts. Please note that two key genes, IL1RL2 and IL33, were consistently up-regulated with about 20 Fold-Change.

**Fig 4:**
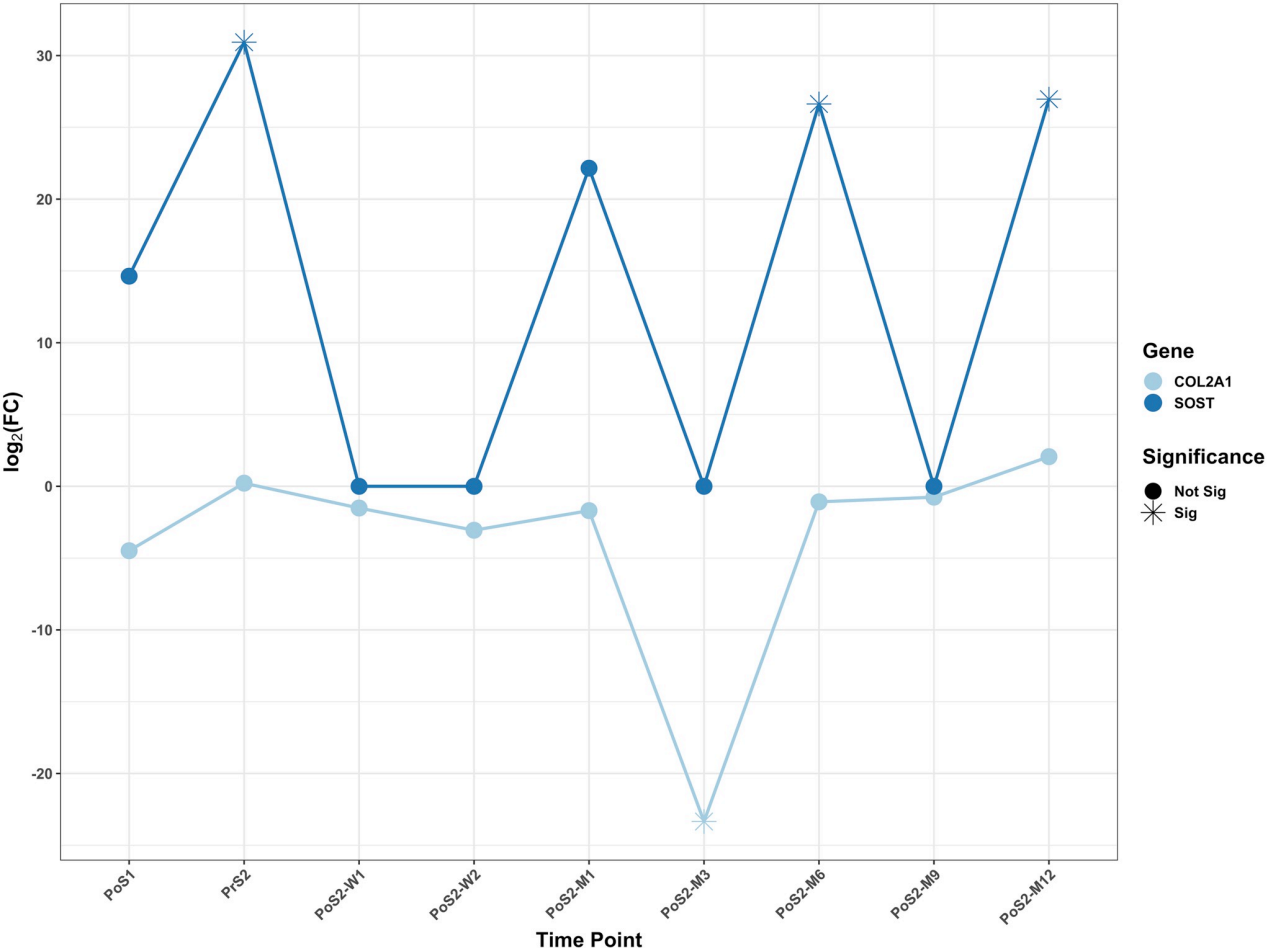
A scattergram showing the significantly differentially expressed genes annotated to bone remodeling-related GO processes. Two genes, SOST and COL2A1, were identified. The SOST exhibited more of the cyclic nature of up-regulation, while COL2A1 was significant at a single time point, at three months, post-surgery 2.

**Fig 5:**
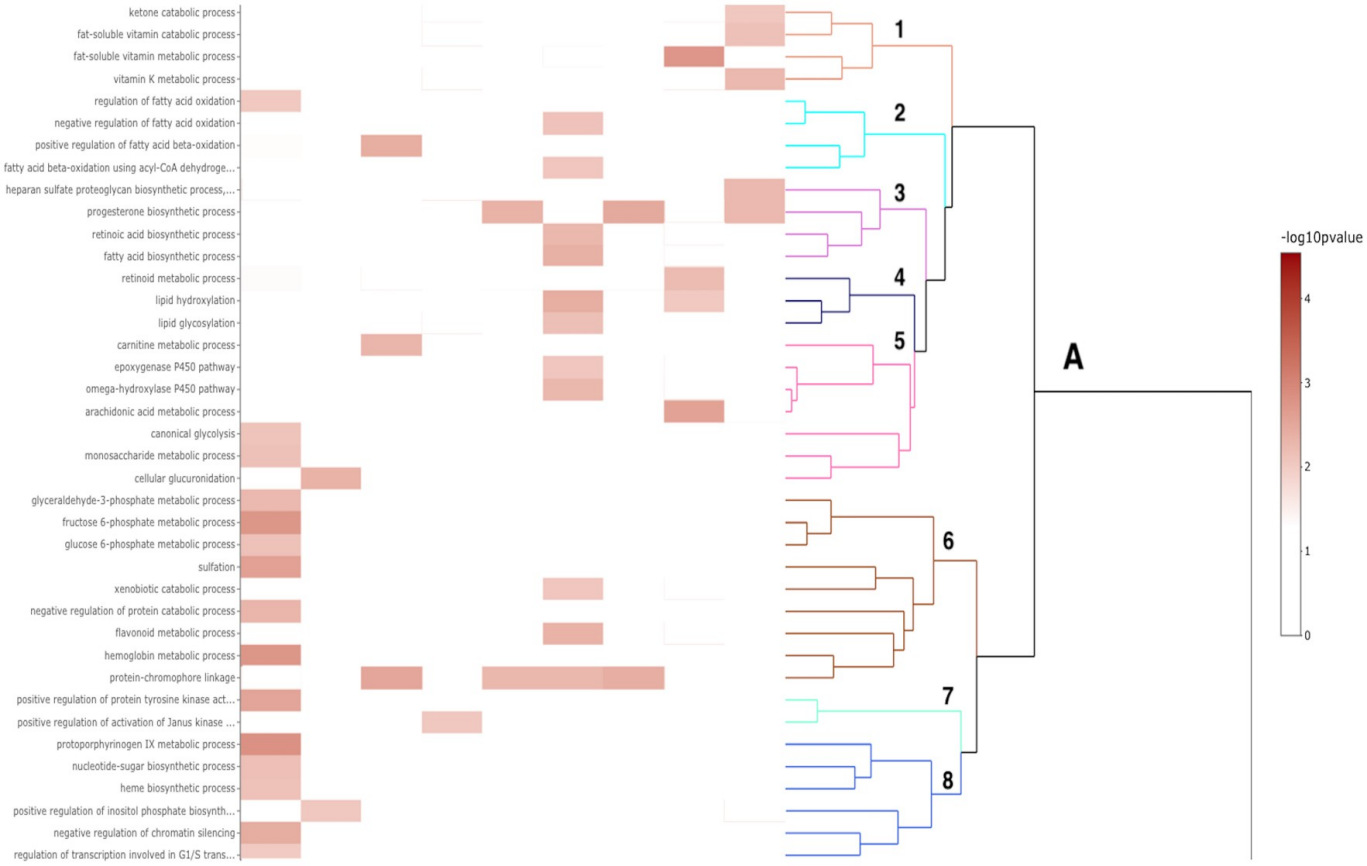
A heat-map showing the clustering of GO biological process terms. Branch A refers to the first main branch for hierarchical clustering based on the semantic similarity between GO terms. Clusters are labeled 1–8, starting at the top. The color of the cell corresponds to the associated p-value from the enrichment analysis.

**Fig 6:**
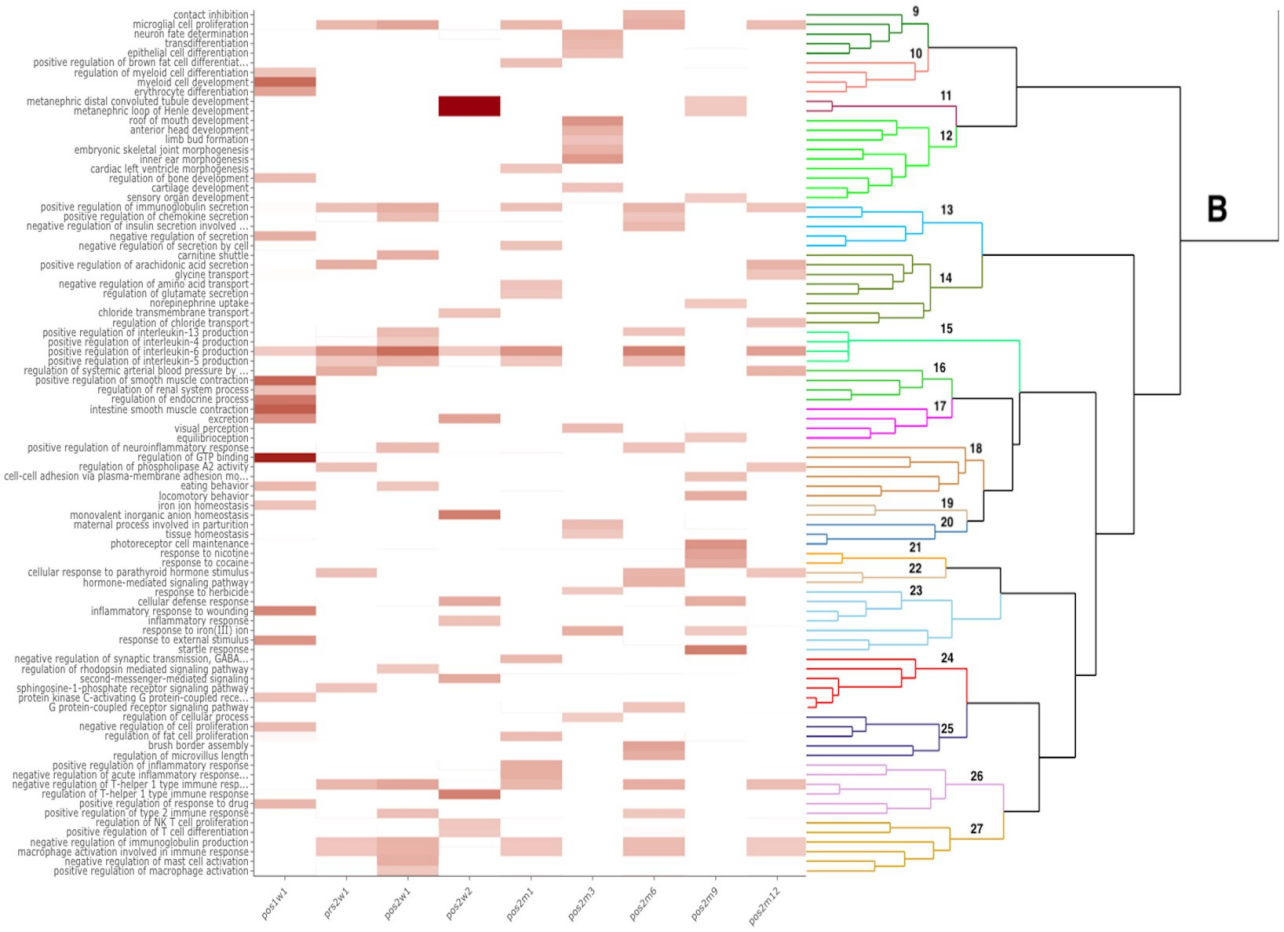
A heat map showing the clustering of GO biological process terms. Branch B refers to the second main branch identified when hierarchical clustering was performed based on the semantic similarity between GO terms. Clusters are labeled 9–27, starting at the top. The color of the cell corresponds to the associated p-value from the enrichment analysis. Multiple processes related to inflammation and bone-remodeling were identified at multiple time points throughout the study.

The majority of Differentially Expressed Genes (DEGs) identified (Table 3) were protein-coding genes. Some non-coding genes were identified, which included lncRNA, miRNA, snRNA, and snoRNA gene types. The DEGs highlighted in this section had a log fold-change (*log*_2_*FC*) less than -1 or greater than 1 and were grouped into inflammation (Fig 3) and bone-remodeling (Fig 4) categories based on their associated GO annotations. A full list of DEGs identified with their associated time point is included in S1 Table. Over the course of the study, 27 clusters were identified from the enrichment analysis (Figs 5 and 6).

**Table 3:** Summary of differentially expressed genes with corresponding HGB content prior to removal of HBA1, HBA2, HBB, HBD, HBG1, HBG2, HBE1, HBM, HBQ1, HBZ transcriptomes bioinformatically, using Ensembl annotations. On average, ~ 29% of reads were related to HGB.

Time Point	HGB (%)	Down-Regulated	Up-Regulated	Total
PoS1	33.6	40	71	111
PrS2	29.2	21	17	38
PoS2-W1	34.8	19	19	38
PoS2-W2	39.7	44	19	63
PoS2-M1	28.9	22	16	38
PoS2-M3	28.9	22	22	44
PoS2-M6	24.2	14	18	32
PoS2-M9	21.3	62	14	76
PoS2-M12	23.5	24	18	42

Most DEGs were only detected at PoS1 immediately after the first surgery. However, a handful of DEGs was identified at later time points and annotated to inflammation-related GO terms: GRB7, IGHD2-2, IGKV1-16, IL12B, IL1RL2, IL33, NPY5R, PRG2, AND SEMA3 (Fig 3). Two genes, IL1RL2 and IL33, continued to be significant at most time points following the first surgery. Multiple GO terms involved in the inflammatory response and enriched at multiple time points were identified in clusters 13, 15, and 27 (Fig 5).

Two DEGs involved in bone remodeling were identified: COL2A1 and SOST (Fig 6). SOST was found to be significant at multiple time points. From the enrichment analysis, the GO term, *regulation of bone development*, was significant at PoS1.

### qPCR validation

**Fig 7:**
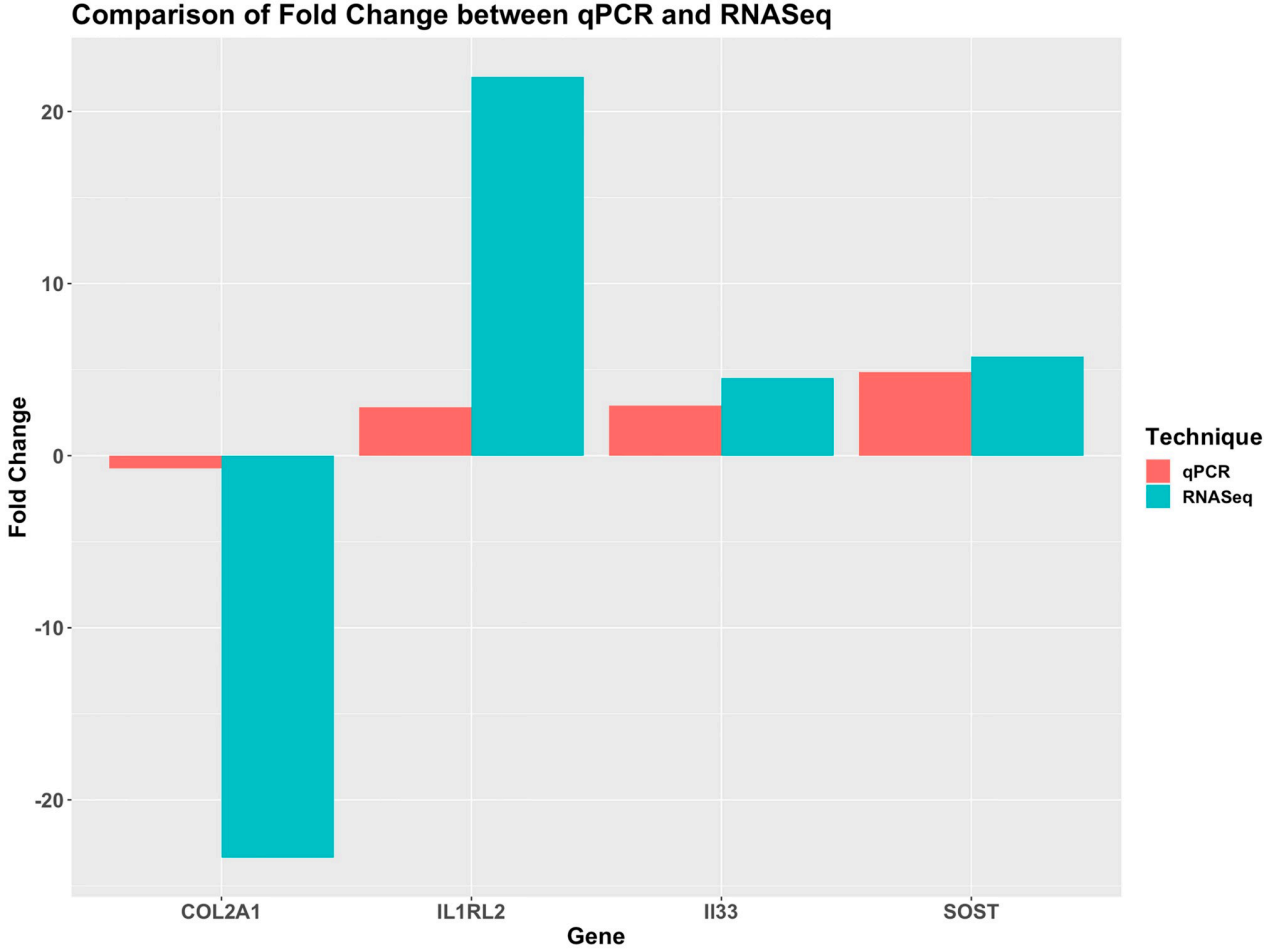
A bar chart comparing the fold change of qPCR results to the log-fold-change from RNA-sequencing. A similar trend is observed between the qPCR and RNA-sequencing results.

The qPCR data for fold-change values followed a similar trend to the RNA-seq fold-change values with a correlation coefficient of 0.75 (Fig 7).

## Discussion

In this longitudinal study, the systemic expression of RNA from whole blood gene expression was surveyed in a ten-patient cohort, in which all patients were fitted with a percutaneous OI prosthetic system. Repeated measure designs, such as the one employed by this study, decrease the amount of variability experienced between measures by having the patients serve as their own baseline. Overall, the results showed at least four circulating genes of interest that could help to understand early bone remodeling and inflammation: IL1RL2, IL33, COL2A1, and SOST.

Multiple DEGs, annotated to inflammation-related GO terms, were identified from the enrichment analysis. Most DEGs were only significant at PoS1. However, two genes, IL33 and IL1RL2 were consistently up-regulated following the first procedure. Interestingly, IGHD2-2 was significantly down-regulated at time points when IL33 returned to baseline (PoS2-M3 and PoS2-M9), the reason for this finding is not readily apparent, but is currently being investigated. IL-33 and IL1RL2 (the latter which serves as a receptor for IL36) are actively involved in the inflammatory process. In mice, IL-33 has been shown to be involved in bacterial defense by inducing nitric oxide production [32]. In another study, inflammation was significantly reduced in IL-36^-/-^ mice who were exposed to *Staphylococcus aureus* (*S*. *aureus*) [33]. Interestingly, our previously reported study investigating the microbiome of the stoma in these same patients identified an increase in the relative proportions of *Streptococcus* and *Staphylococcus* bacteria over time [19]. This result indicates that the periprosthetic tissues may be increasingly exposed to colonization by *S*. *aureus*, and that the elevated levels of IL33 and IL1RL2 may contribute to providing local immunity against superficial infection. Conversely, in the absence of a matured stoma and a skin seal against the implant, it is possible that the sustained upregulation of IL-33 and IL1RL2 may indicate increased ingress of potentially pathogenic but colonizing bacteria into the peri-prosthetic tissue, albeit in the absence of clinical infection. It is also possible that increased systemic expression of IL-33 and IL1RL2 could be attributed to the patient’s adaptive immune responses to minimize infection associated with the infiltration of bacteria into the periprosthetic tissue from the percutaneous stoma. However, further investigation is warranted to confirm these assumptions.

COL2A1 is typically expressed in cartilaginous tissue and is involved in the production of collagen type 2 extracellular matrix [34]. For this gene, we observed a down-regulation of over 20-fold in the circulating blood at PoS2-M3. Immediately following the second surgery, our patients were able to load their endoprosthesis. Post-loading, we expect the host bone to undergo accelerated remodeling as supported by our ovine preclinical data, which showed an accelerated bone remodeling period within 3-months of post-implantation [35, 36]. Furthermore, COL2A1 expression has been reported within the local bone tissue during the remodeling process [37]. The question remains as to whether or not the downregulated Col2A1 at 3-months following the second surgery marks the end of the accelerated bone remodeling period. Although COL2A1 is not directly related to the bone matrix, scientists increasingly recognize the emerging role of chondrocyte-to-osteoblast transdifferentiation in the endochondral ossification process [38]. Moreover, in a mouse dental implant study, COL2A1 was upregulated during the osseointegration process within the local oral tissue [39]. Perhaps, this transdifferentiation is also the molecular pathway plays a role in the osseointegration of OI implants. This concept also warrants further investigation.

In the presented dataset, SOST expression levels were significant and cyclic following the first surgery. This may indicate control of local bone resorption/deposition and possibly ongoing changes in the rate of osseointegration. Thus, SOST is another potential gene of interest. Although SOST mRNA is secreted primarily by osteocytes, very small quantities are reported to be secreted by basophils, eosinophils, and neutrophils [40]. The protein encoded by the SOST gene, sclerostin, belongs to the family of bone morphogenetic protein (BMP) antagonists and is known to regulate bone turnover, i.e., negatively regulate bone formation [41]. Sclerostin is also involved in regulating osteocytes, which are major mechano-sensors in the bone. In our study, SOST expression was initially upregulated between the first and second surgeries. This may indicate bone resorption due to nonloading during this period which is when patients are advised to not load their implant limb. This phase was followed by decreased SOST expression, with loading between second surgery and PoS2-M3. A similar result was observed, in a mouse study, within the local tissue [39]. Our previous *in vivo* ovine studies supported this observation in which the growth of new bone into the cancellous titanium structure at the endosteal interface occurred very rapidly but required over 12-months to reach a steady state [35, 36, 42]. It is also known that differentiating osteoblasts at the end of the bone deposition period are known to transition into osteocytes. Thus, at the end of the accelerated bone remodeling period, this transitioning phase may have resulted in the observed SOST overexpression. Furthermore, multiple studies in the literature highlight the importance of sclerostin in bone remodeling and development. In a SOST knockout mouse study, Shu and his coworkers have shown that inhibition of this gene improved implant fixation in osteoporotic bone [43]. Other authors have shown that when the osseointegrated implant was loaded, local SOST expression was downregulated [44]. Thus, SOST serves as another serological gene candidate for studying osseointegration and bone-resorption following any orthopedic implantation surgery.

The qPCR fold-change values of these four genes, in a subset of patients, followed a similar trend when compared to the RNA-seq log-fold-change values with a correlation of 0.75 validating our DEG analyses data.

The current study is not without limitations. Although the samples had, on average, ~30 million reads, this depth of sequencing coverage may be inadequate with the removal of HGB-related counts and the presence of multiple different cell types contained in the blood. To alleviate these issues in future studies, one may incorporate a globin mRNA depletion step to allow for wider read coverage and/or increase the depth of coverage per sample. Another limitation included the small number of patients enrolled in the study. Ten patients were initially enrolled, including two of whom which experienced implant failures. Additionally, it is possible that the statistical model used for identifying DEGs may suffer from overfitting by including multiple surrogate variables in the model. The majority of DEGs were detected at PoS1, and most genes approached their initial baseline expression shortly following the second surgery. However, a handful of inflammatory-related genes continued to be significant at twelve months following the Stage 2 Surgery. It is impossible, with the current study design, to determine if these genes would return to baseline or continue to be up-regulated given additional time. Although four genes were validated using qPCR, further protein expression validation is warranted. Such assays may involve collecting local stomal exudate (which can, but not always, be present clinically) or local tissues where respective proteins may have been translated rather than in the blood. Future animal studies and human trials will be used to confirm these findings.

In conclusion, this preliminary study of whole-blood RNA-seq from 10 transfemoral amputees, who had previously undergone percutaneous osseointegrated skeletal docking of a prosthetic lower limb, uncovered two major results 1) Two inflammatory-related genes (IL33 and IL1RL2) were found to be differentially expressed by 20-fold. These genes may serve as important biomarkers in the serial assessment of stomal wound healing. 2) Two other biomarkers related to bone remodeling (COL2A1 and SOST) were identified. If verified by future studies, these four genes may serve as markers for predicting optimal bone remodeling and stomal tissue healing following the percutaneous OI implantation procedure.

## Supporting information

S1 Table Combined list of all differentially expressed genes identified. (XLSX)

## References

[1] Al Muderis M, Khemka A, Lord SJ, Van de Meent H, Frölke JPM. Safety of Osseointegrated Implants for Transfemoral Amputees: A Two-Center Prospective Cohort Study. The Journal of Bone and Joint Surgery. 2016;98(11):900–9. doi: 10.2106/JBJS.15.00808 27252434

[2] Brånemark R, Berlin Ö, Hagberg K, Bergh P, Gunterberg B, Rydevik B. A novel osseointegrated percutaneous prosthetic system for the treatment of patients with transfemoral amputation: A prospective study of 51 patients. The Bone & Joint Journal. 2014;96-B(1):106–13. doi: 10.1302/0301-620X.96B1.31905 24395320

[3] Juhnke D-L, Beck JP, Jeyapalina S, Aschoff HH. Fifteen years of experience with Integral-Leg-Prosthesis: Cohort study of artificial limb attachment system. J Rehabil Res Dev. 2015;52(4):407–20. doi: 10.1682/JRRD.2014.11.0280 26348827

[4] Al Muderis MM, Lu WY, Li JJ, Kaufman K, Orendurff M, Highsmith MJ, et al. Clinically Relevant Outcome Measures Following Limb Osseointegration; Systematic Review of the Literature. Journal of Orthopaedic Trauma. 2018;32(2):e64–e75. doi: 10.1097/BOT.0000000000001031 29373379

[5] Lundberg M, Hagberg K, Bullington J. My prosthesis as a part of me: a qualitative analysis of living with an osseointegrated prosthetic limb. Prosthet Orthot Int. 2011;35(2):207–14. doi: 10.1177/0309364611409795 21697203

[6] Lee TC, Taylor D. Bone remodelling: should we cry Wolff? Irish Journal of Medical Science. 1999;168:102–5. doi: 10.1007/BF02946474 10422387

[7] Stein H, D’Ambrosia R. Wolff’s Law Decoded. Orthapedics. 2008;31:213. doi: 10.3928/01477447-20080301-38 18351040

[8] Frost HM. A 2003 Update of Bone Physiology and Wolff’s Law for Clinicians. Angle Orthodontist. 2004;74(1):13. 10.1043/0003-3219(2004)074<0003:AUOBPA>2.0.CO;2 15038485

[9] Potter BK. CORR Insights(R): What Are the Risk Factors for Mechanical Failure and Loosening of a Transfemoral Osseointegrated Implant System in Patients with a Lower-limb Amputation? Clin Orthop Relat Res. 2022. Epub 2022/01/13. doi: 10.1097/CORR.0000000000002112 . 35020624 PMC8923605

[10] Tsikandylakis G, Berlin Ö, Brånemark R. Implant Survival, Adverse Events, and Bone Remodeling of Osseointegrated Percutaneous Implants for Transhumeral Amputees. Clinical Orthopaedics and Related Research^®^. 2014;472(10):2947–56. doi: 10.1007/s11999-014-3695-6 24879569 PMC4160502

[11] Tillander J, Hagberg K, Hagberg L, Brånemark R. Osseointegrated Titanium Implants for Limb Prostheses Attachments: Infectious Complications. Clinical Orthopaedics and Related Research^®^. 2010;468(10):2781–8. doi: 10.1007/s11999-010-1370-0 20473597 PMC2939339

[12] Stenlund P, Trobos M, Lausmaa J, Branemark R, Thomsen P, Palmquist A. Effect of load on the bone around bone-anchored amputation prostheses. J Orthop Res. 2017;35(5):1113–22. Epub 2016/06/25. doi: 10.1002/jor.23352 . 27341064

[13] Tillander J, Hagberg K, Berlin Ö, Hagberg L, Brånemark R. Osteomyelitis Risk in Patients With Transfemoral Amputations Treated With Osseointegration Prostheses. Clinical Orthopaedics and Related Research^®^. 2017;475(12):3100–8. doi: 10.1007/s11999-017-5507-2 28940152 PMC5670076

[14] Holt BM, Betz DH, Ford TA, Beck JP, Bloebaum RD, Jeyapalina S. Pig dorsum model for examining impaired wound healing at the skin-implant interface of percutaneous devices. J Mater Sci: Mater Med. 2013;24(9):2181–93. doi: 10.1007/s10856-013-4975-5 23832453 PMC3770289

[15] Maglio M, Salamanna F, Brogini S, Borsari V, Pagani S, Nicoli Aldini N, et al. Histological, Histomorphometrical, and Biomechanical Studies of Bone-Implanted Medical Devices: Hard Resin Embedding. Biomed Res Int. 2020;2020:1804630. Epub 2020/05/19. doi: 10.1155/2020/1804630 . 32420323 PMC7201441

[16] Lin Z, Rios HF, Volk SL, Sugai JV, Jin Q, Giannobile WV. Gene expression dynamics during bone healing and osseointegration. J Periodontol. 2011;82(7):1007–17. Epub 2010/12/15. doi: 10.1902/jop.2010.100577 . 21142982 PMC3399909

[17] Lenneras M, Palmquist A, Norlindh B, Emanuelsson L, Thomsen P, Omar O. Oxidized Titanium Implants Enhance Osseointegration via Mechanisms Involving RANK/RANKL/OPG Regulation. Clin Implant Dent Relat Res. 2015;17 Suppl 2:e486–500. Epub 2014/12/24. doi: 10.1111/cid.12276 . 25536123

[18] Vlot MC, den Heijer M, de Jongh RT, Vervloet MG, Lems WF, de Jonge R, et al. Clinical utility of bone markers in various diseases. Bone. 2018;114:215–25. doi: 10.1016/j.bone.2018.06.011 29920402

[19] Beck JP, Grogan M, Bennett BT, Jeyapalina S, Agarwal J, Bartow‐McKenney C, et al. Analysis of the Stomal Microbiota of a Percutaneous Osseointegrated Prosthesis: A Longitudinal Prospective Cohort Study. Journal of Orthopaedic Research. 2019;37(12):2645–54. doi: 10.1002/jor.24421 31317568

[20] Ewels P, Magnusson M, Lundin S, Käller M. MultiQC: summarize analysis results for multiple tools and samples in a single report. Bioinformatics. 2016;32(19):3047–8. doi: 10.1093/bioinformatics/btw354 27312411 PMC5039924

[21] Andrews S. FastQC. FastQC.

[22] Dobin A, Davis CA, Schlesinger F, Drenkow J, Zaleski C, Jha S, et al. STAR: ultrafast universal RNA-seq aligner. Bioinformatics. 2013;29(1):15–21. doi: 10.1093/bioinformatics/bts635 23104886 PMC3530905

[23] Liao Y, Smyth GK, Shi W. featureCounts: an efficient general purpose program for assigning sequence reads to genomic features. Bioinformatics. 2014;30(7):923–30. doi: 10.1093/bioinformatics/btt656 24227677

[24] Field LA, Jordan RM, Hadix JA, Dunn MA, Shriver CD, Ellsworth RE, et al. Functional identity of genes detectable in expression profiling assays following globin mRNA reduction of peripheral blood samples. Clinical Biochemistry. 2007;40(7):499–502. doi: 10.1016/j.clinbiochem.2007.01.004 17303101

[25] Love MI, Huber W, Anders S. Moderated estimation of fold change and dispersion for RNA-seq data with DESeq2. Genome Biology. 2014;15(12). doi: 10.1186/s13059-014-0550-8 25516281 PMC4302049

[26] Leek JT. svaseq: removing batch effects and other unwanted noise from sequencing data. Nucleic Acids Research. 2014;42(21):e161–e. doi: 10.1093/nar/gku864 25294822 PMC4245966

[27] Brionne A, Juanchich A, Hennequet-Antier C. ViSEAGO: a Bioconductor package for clustering biological functions using Gene Ontology and semantic similarity. BioData Mining. 2019;12(1):16. doi: 10.1186/s13040-019-0204-1 31406507 PMC6685253

[28] The Gene Ontology Consortium. The Gene Ontology Resource: 20 years and still GOing strong. Nucleic Acids Research. 2019;47(D1):D330–D8. doi: 10.1093/nar/gky1055 30395331 PMC6323945

[29] Alexa A, Rahnenfuhrer J, Lengauer T. Improved scoring of functional groups from gene expression data by decorrelating GO graph structure. Bioinformatics. 2006;22(13):1600–7. doi: 10.1093/bioinformatics/btl140 16606683

[30] Wang JZ, Du Z, Payattakool R, Yu PS, Chen CF. A new method to measure the semantic similarity of GO terms. Bioinformatics. 2007;23(10):1274–81. doi: 10.1093/bioinformatics/btm087 17344234

[31] Langfelder P, Zhang B, Horvath S. Dynamic Tree Cut: in-depth description, tests and applications.11.

[32] Li C, Li H, Jiang Z, Zhang T, Wang Y, Li Z, et al. Interleukin-33 Increases Antibacterial Defense by Activation of Inducible Nitric Oxide Synthase in Skin. PLoS Pathog. 2014;10(2):e1003918. doi: 10.1371/journal.ppat.1003918 24586149 PMC3930573

[33] Liu H, Archer NK, Dillen CA, Wang Y, Ashbaugh AG, Ortines RV, et al. Staphylococcus aureus Epicutaneous Exposure Drives Skin Inflammation via IL-36-Mediated T Cell Responses. Cell Host & Microbe. 2017;22(5):653–66.e5. doi: 10.1016/j.chom.2017.10.006 29120743 PMC5774218

[34] Okazaki K, Sandell LJ. Extracellular Matrix Gene Regulation. Clinical Orthopaedics and Related Research. 2004;427:S123–S8. doi: 10.1097/01.blo.0000144478.51284.f3 15480054

[35] Jeyapalina S, Beck JP, Bachus KN, Bloebaum RD. Cortical Bone Response to the Presence of Load-Bearing Percutaneous Osseointegrated Prostheses. Anat Rec. 2012;295(9):1437–45. doi: 10.1002/ar.22533 22807281

[36] Jeyapalina S, Beck JP, Bloebaum RD, Bachus KN. Progression of Bone Ingrowth and Attachment Strength for Stability of Percutaneous Osseointegrated Prostheses. Clinical Orthopaedics and Related Research^®^. 2014;472(10):2957–65. doi: 10.1007/s11999-013-3381-0 24258685 PMC4160472

[37] Thorfve A, Lindahl C, Xia W, Igawa K, Lindahl A, Thomsen P, et al. Hydroxyapatite coating affects the Wnt signaling pathway during peri-implant healing in vivo. Acta Biomaterialia. 2014;10(3):1451–62. doi: 10.1016/j.actbio.2013.12.012 24342040

[38] Aghajanian P, Mohan S. The art of building bone: emerging role of chondrocyte-to-osteoblast transdifferentiation in endochondral ossification. Bone Res. 2018;6:19. Epub 2018/06/22. doi: 10.1038/s41413-018-0021-z . 29928541 PMC6002476

[39] Biguetti CC, Cavalla F, Silveira EM, Fonseca AC, Vieira AE, Tabanez AP, et al. Oral implant osseointegration model in C57Bl/6 mice: microtomographic, histological, histomorphometric and molecular characterization. J Appl Oral Sci. 2018;26:e20170601. Epub 2018/06/14. doi: 10.1590/1678-7757-2017-0601 . 29898187 PMC5963915

[40] Weivoda MM, Youssef SJ, Oursler MJ. Sclerostin expression and functions beyond the osteocyte. Bone. 2017;96:45–50. doi: 10.1016/j.bone.2016.11.024 27888056 PMC5328839

[41] Delgado-Calle J, Sato AY, Bellido T. Role and mechanism of action of sclerostin in bone. Bone. 2017;96:29–37. doi: 10.1016/j.bone.2016.10.007 27742498 PMC5328835

[42] Jeyapalina S, Beck JP, Drew A, Bloebaum RD, Bachus KN. Variation in bone response to the placement of percutaneous osseointegrated endoprostheses: A 24-month follow-up in sheep. PLoS ONE. 2019;14(10):e0221850. doi: 10.1371/journal.pone.0221850 31652276 PMC6814231

[43] Shu R, Ai D, Bai D, Song J, Zhao M, Han X. The effects of SOST on implant osseointegration in ovariectomy osteoporotic mice. Archives of Oral Biology. 2017;74:82–91. doi: 10.1016/j.archoralbio.2016.11.012 27918899

[44] Jing D, Tong S, Zhai M, Li X, Cai J, Wu Y, et al. Effect of low-level mechanical vibration on osteogenesis and osseointegration of porous titanium implants in the repair of long bone defects. Scientific Reports. 2015;5(1):17134. doi: 10.1038/srep17134 26601709 PMC4658533

